# Differential Phagocytosis of White versus Opaque *Candida albicans* by *Drosophila* and Mouse Phagocytes

**DOI:** 10.1371/journal.pone.0001473

**Published:** 2008-01-23

**Authors:** Matthew B. Lohse, Alexander D. Johnson

**Affiliations:** 1 Department of Biochemistry and Biophysics, University of California at San Francisco, San Francisco, California, United States of America; 2 Department of Microbiology and Immunology, University of California at San Francisco, San Francisco, California, United States of America; Theodor-Boveri-Institut fur Biowissenschaften, Wurzburg, Germany

## Abstract

The human fungal pathogen *Candida albicans* resides asymptomatically in the gut of most healthy people but causes serious invasive diseases in immunocompromised patients. Many *C. albicans* strains have the ability to stochastically switch between distinct white and opaque cell types, but it is not known with certainty what role this switching plays in the physiology of the organism. Here, we report a previously undescribed difference between white and opaque cells, namely their interaction with host phagocytic cells. We show that both *Drosophila* hemocyte-derived S2 cells and mouse macrophage-derived RAW264.7 cells preferentially phagocytose white cells over opaque cells. This difference is seen both in the overall percentage of cultured cells that phagocytose white versus opaque *C. albicans* and in the average number of *C. albicans* taken up by each phagocytic cell. We conclude that susceptibility to phagocytosis by cells of the innate immune system is an important distinction between white and opaque *C. albicans*, and propose that one role of switching from the prevalent white form into the rarer opaque form may be to allow *C. albicans* to escape phagocytosis.

## Introduction

Many strains of the commensal yeast *Candida albicans* undergo rare, stochastic switching between two distinct types of cells, white and opaque [1]. The opaque phase was first noted due to its distinct colony morphology; at the single cell level, opaque cells are elongated, while white cells are nearly spherical [reviewed by 2]–[4]. Approximately 400 genes are differentially expressed in white versus opaque cells, resulting in a wide variety of phenotypic differences [5], [6]. For example, white and opaque cells differ markedly in their ability to mate: mating between opaque cells of the opposite mating type is at least 10^6^ times more efficient than mating between white cells [7]. White and opaque cells also differ in their suitability for different host environments; opaque cells appear better at colonizing the skin while white cells appear better suited to the bloodstream and internal environments [8]–[10].

In addition to the differences discussed above, white and opaque cells appear to interact differentially with the host immune system. Kolotila *et al*., 1990, reported that opaque cells were more susceptible than white cells to killing by oxidative stress and by neutrophils [11]. These stresses were observed to increase the frequency of switching to the opaque phase, raising the possibility that Candida cells may respond to the innate immune system by modulating their rates of white-opaque switching [11]. More recently, Geiger *et al*. 2004 showed that leukocytes respond differently to white and opaque cells. Both types of cells induce chemokinesis in leukocytes, but only white cells release a chemoattractant promoting chemotaxis towards a *C. albicans* source [12]. These results suggest that opaque cells may be less “visible” to the innate immune system, an idea which could have important implications for pathogenesis, commensalism, and mating.

In this paper, we examine whether phagocytic cells derived from the innate immune system differ in their ability to phagocytose white and opaque cells. We carried out this analysis first using *Drosophila melanogaster* S2 cells, a hemocyte-derived line. The use of *D. melanogaster* S2 cells for studying aspects of the innate immune response to *C. albicans* and other pathogens has been documented by numerous studies [13]–[15]. We also examined differences in the response of *Mus musculus* RAW 264.7 cells, a macrophage-derived line, to *C. albicans* white and opaque cells. The use of this cell line to study the innate immune response to pathogens, including *C. albicans*, is also well-documented [see, for example, 16]. In both the fly and mouse systems, we found that opaque cells were phagocytosed at significantly lower rates than were white cells. These results reveal an additional difference between white and opaque cells, one with implications for pathogenesis, and provide independent evidence that opaque cells are “less visible” than white cells to the innate immune system.

## Results

### Differential phagocytosis of white and opaque *C. albicans* by S2 cells

**Figure 1:**
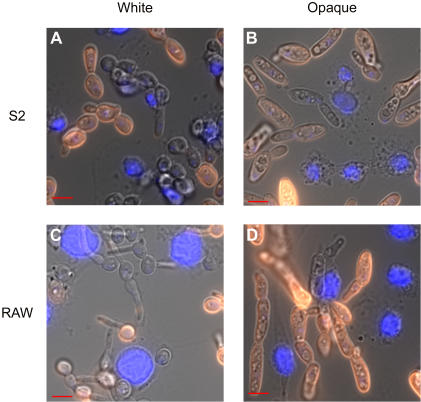
Phagocytosis of white and opaque *C. albicans* by *D. melanogaster* S2 and *M. musculus* RAW cells. White (A,C) or opaque (B,D) *C. albicans* cells were co-incubated with S2 cells for 3.5 hours (A,B) or RAW cells for 1 hour (C,D), lightly fixed with formaldehyde, stained with rabbit anti-Candida and Cy3-labeled anti-rabbit antibodies. Cells were then stained with a DAPI solution (blue) to localize S2 or RAW cells. *C. albicans* cells that were not phagocytosed appear orange in these figures.

Recent studies have established *D. melanogaster* as a model system for studying many human pathogens, including *C. albicans*. Several RNAi screens have revealed a core set of genes required for phagocytosis of many microbes as well as additional genes needed specifically for individual pathogens [13], [15], [17]–[19]. These studies have also revealed differences in the efficiency of phagocytosis among fungi. For example, Stroschein-Stevenson *et al*., 2006 showed that *C. albicans* is much more efficiently phagocytosed than the non-pathogenic yeast *Saccharomyces cerevisiae* [15]. To compare phagocytosis of *C. albicans* white and opaque cells, *C. albicans* cells were co-incubated with S2 cells and after variable time intervals the samples were lightly formaldehyde fixed, and phagocytosis was scored using immunofluoresence. For this analysis, the fixed cells were stained with rabbit anti-*Candida* and anti-rabbit Cy3-labeled antibodies followed by DAPI. Since membranes were not permeabilized until after antibody staining, internalized *C. albicans* lack Cy3 and are visually distinguishable from the cells that were not phagocytosed. This assay, optimized to study *C. albicans* white cells [15] (FIG. 1A), also efficiently monitors opaque cell phagocytosis (FIG. 1B).

**Figure 2:**
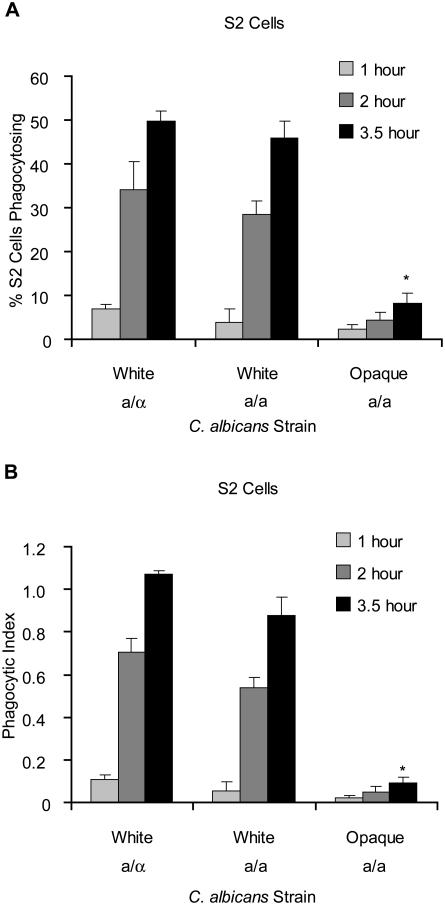
*D. melanogaster* S2 cells more efficiently phagocytose white than opaque *C. albicans*. (A) *D. melanogaster* S2 cells were co-incubated with white or opaque *C. albicans* for 1, 2, or 3.5 hours, fixed, and stained as described in the Materials and Methods. The number of S2 cells phagocytosing one or more *C. albicans* cells was determined. (B) For the same sets of S2 cells, the number of *C. albicans* cells phagocytosed by S2 cells was quantified and the total number of *C. albicans* cells phagocytosed divided by the number of S2 cells scored, referred to as the phagocytic index, was plotted. Values plotted are the averages from four data sets from a given day and error bars represent the standard deviation. 100 S2 cells were counted for each data set. For the 3.5 hour time point, statistical significance of differences from the white a/a strain was evaluated using a t-test assuming unequal variance, sets with p<.001 are marked with an asterisk.

Using this assay, we compared the ability of S2 cells to phagocytose white and opaque *C. albicans* cells of exactly the same genotype. S2 cells were co-incubated with either white or opaque *C. albicans* at a multiplicity of infection (MOI) of 5. After various time periods, the samples were fixed and stained as described in Materials and Methods. From each infection, four separate sets of 100 S2 cells were scored for phagocytosis of *C. albicans* cells and the values averaged. After a 2 hour co-incubation, approximately 5-fold more S2 cells had taken up at least one white cell than had taken up at least one opaque cell, even though the S2 cells were exposed to equal numbers of *C. albicans* cells (FIG. 2A). Similar differences were also seen after 3.5 hours of co-incubation. This difference was independent of mating type: **a**/**a** whites were phagocytosed similarly to **a**/α (FIG. 2B) and α/α whites, while **a**/**a** opaques were phagocytosed similarly to α/α opaques (data not shown). This difference between whites and opaques repeated on multiple days and in multiple *C. albicans* strain backgrounds despite fluctuations in overall levels of phagocyosis. Previous studies have shown that S2 cells preferentially phagocytose *C. albicans* white cells over non-pathogenic *S. cerevisiae* [15]; the level of opaque cell phagocytosis we observed in this study is approximately equal to that of *S. cerevisisae* (data not shown).

In addition to scoring differences in the percentages of S2 cells that phagocytosed white versus opaque cells, we examined the total number of *C. albicans* cells phagocytosed by each set of 100 S2 cells scored. Since this value incorporates both differences in the number of S2 cells that had taken up at least one *C. albicans* cell and the average number of *C. albicans* cells phagocytosed per S2 cell, it more accurately reflects differences in the efficiency of phagocytosis. The value for white *C. albicans* cells is 5 to 10-fold greater after 2 hours and approximately 10-fold greater after 3.5 hours than the equivalent value for opaque cells (FIG. 2B).

**Figure 3:**
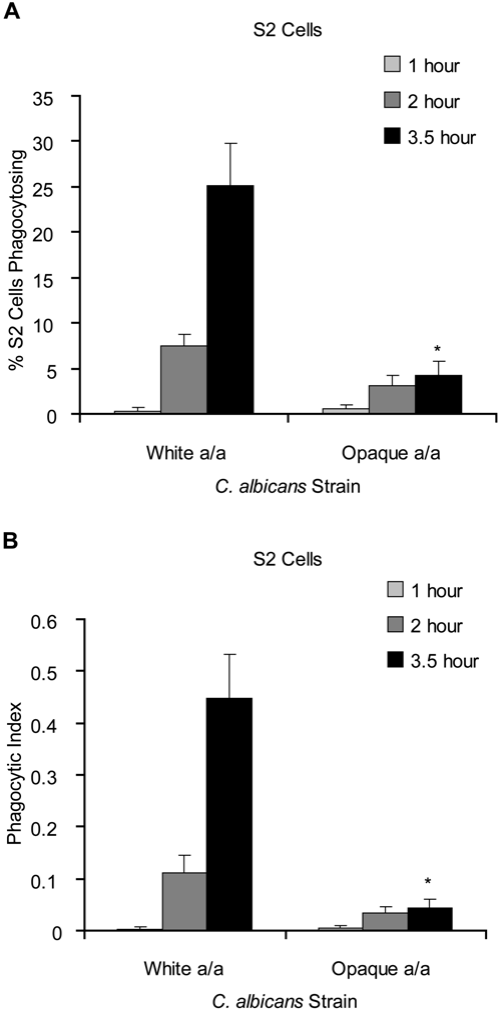
S2 cells preferentially phagocytose white *C. albicans* from a mixed white-opaque population. (A) *D. melanogaster* S2 cells were co-incubated with equal numbers of white and opaque *C. albicans* for 1, 2, and 3.5 hours. The number of S2 cells phagocytosing one or more *C. albicans* cells was determined. (B) For the same S2 cells, the number of *C. albicans* cells phagocytosed was quantified and the total number of *C. albicans* cells phagocytosed divided by the number of S2 cells scored, referred to as the phagocytic index, was plotted. 100 S2 cells were counted for each data set; values reflect the average of six data sets. For the 3.5 hour time point, statistical significance of differences from the a/a whites was determined using a t-test and differences with p<.001 are marked with an asterisk.

We independently confirmed the preference of S2 cells for white over opaque cells by presenting the S2 cells with mixed populations of white and opaque *C. albicans* cells. In the experiments shown in Figure 3, S2 cells were presented with a 50∶50 mixture of white and opaque cells whose combined cell number was equal to the number of pure white or opaque cells used in the previous assays. White and opaque cells were easily distinguished microscopically and were counted separately using the described Cy3 antibody based strategy described in Materials and Methods. As before, sets of 100 S2 cells were counted at a time, but in this case six data sets were collected for each experiment and combined to calculate average values and standard deviations. When presented with a mixed *C. albicans* culture, S2 cells were 6-fold more likely to have phagocytosed at least one white cell than at least one opaque cell after 3.5 hours (FIG. 3A). Similarly, the number of *C. albicans* cells phagocytosed per S2 cell scored was approximately 10-fold higher for white cells than opaque cells (FIG. 3B). Although most S2 cells that phagocytosed multiple *C. albicans* cells took up either exclusively white or opaque cells, an occasional S2 cell was observed to have phagocytosed a mixture of white and opaque cells. We believe that switching between the white and the opaque phases during the assay is minimal, based on the short duration of the assay as well as plating assays which indicate that the switching rate of *C. albicans* does not change significantly on exposure to S2 cells (data not shown).

### RAW cells are also more efficient at phagocytosing white than opaque *C. albicans*

We next tested whether a mouse macrophage line exhibited the same preference for white cells over opaque cells. We modified the procedure described above to accommodate cultured mammalian cells (see Materials and Methods) and established that the procedure could detect uptake of both white (FIG. 1C) and opaque (FIG. 1D) cells of *C. albicans*. The combination of 10% serum and 37°C temperature needed for growth of the macrophage cell line complicated the experiment at later time points, as *C. albicans* extensively filaments under these conditions. Moreover, opaque cells are not stable during prolonged exposure at 37°C and eventually revert to white cells [20]. For these reasons, we concentrated on early (less than 2 hours) timepoints for this analysis.

**Figure 4:**
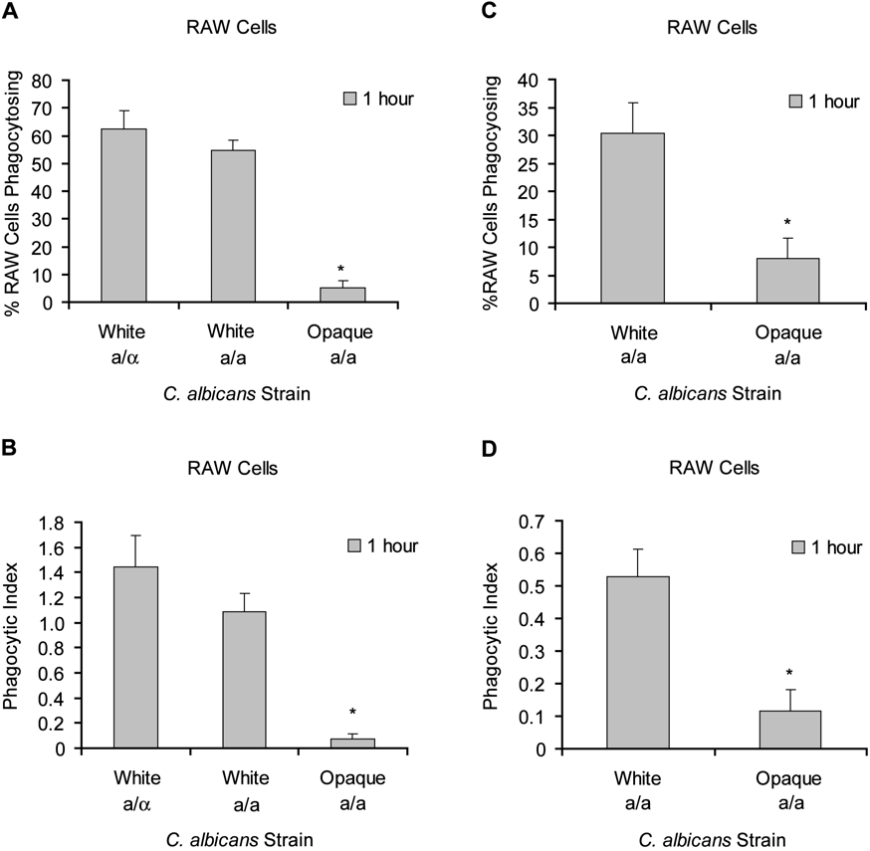
*M. musculus* RAW cells more efficiently phagocytose white than opaque *C. albicans*. (A) *M. musculus* RAW cells were co-incubated with white or opaque *C. albicans* for 1 hour then stained using the same methods as for S2 cells. The number of RAW cells phagocytosing one or more *C. albicans* cells was determined. (B) The number of *C. albicans* cells phagocytosed was quantified and used to calculate and plot the total number of *C. albicans* cells phagocytosed divided by the number of RAW cells scored, referred to as the phagocytic index. (C) *M. musculus* RAW cells were co-incubated with equal numbers of white and opaque *C. albicans* for 1 hour. The number of RAW cells phagocytosing one or more *C. albicans* cells was determined. (D) For the same RAW cells, the number of *C. albicans* cells phagocytosed was quantified and the phagocytic index plotted. 100 RAW cells were counted for each data set and values plotted are the average of four (A,B) or six (C,D) data sets with error bars representing standard deviation. Statistical significance of differences from the a/a whites was determined using a t-test and differences with p<.001 are marked with an asterisk.

After a 1 hour co-incubation, the percentage of RAW cells that had phagocytosed at least one white *C. albicans* was roughly 5 to 10-fold greater than for opaques (FIG. 4A). As with S2 cells, **a**/α and **a**/**a** whites were phagocytosed at similar rates (FIG. 4A) as were equivalent α/α strains (data not shown). We also monitored the total number of *C. albicans* phagocytosed and used this to calculate the number of *C. albicans* cells phagocytosed per RAW cell scored. The value for white cells was approximately 10-fold greater than opaques after one hour (FIG. 4B).

When presented with a mixed population of *C. albicans* cells, the RAW cells showed a trend similar to the results using S2 cells: at 1 hour a given macrophage-derived cell was 4 to 6-fold more likely to have phagocytosed at least one white cell than at least one opaque cell (FIG. 4C). In these mixed experiments, we observed the number of *C. albicans* cells phagocytosed per RAW cell scored was 4 to 8-fold higher for white cells than for opaque cells (FIG. 4D). The majority of RAW cells phagocytosed exclusively white or opaque *C. albicans*, but as with S2 cells there were a small number of cases where a RAW cell was observed to have phagocytosed both cell types.

To determine whether the difference in phagocytosis rates of white and opaque cells reflects a difference in adherence, we modified the assay for use at 4°C, a temperature that allows adherence but blocks phagocytosis (see Materials and Methods). We found that adherence levels were roughly 2 to 3-fold greater for white cells than for opaque cells (data not shown). This difference is significantly lower than the difference in phagocytosis and indicates that, although contributory, adherence is unlikely to be responsible for the entire effect.

## Discussion

White-opaque switching is one of the most enigmatic features of *Candida albicans*. Switching occurs, on average, every 10^3^–10^4^ generations, and the white and opaque forms are each heritable for many generations- until a new switching event occurs. It has been proposed that the heritability of the two states is based on a self-perpetuating feedback loop which is excited in the opaque state but which remains broken in the white state [21]–[23]. Regardless of the exact mechanism, the phenomenon appears to be epigenetic, that is, switching from the white form to the opaque form and back occurs without any changes in the primary DNA sequence of the *Candida* genome.

Despite having identical genomes, white and opaque cells show enormous phenotypic differences. The two types of cells are easily distinguished under the microscope and each gives rise to a specific type of colony easily distinguished by the naked eye [for reviews see 2]–[4]. The two types of cells appear to favor different niches in the host: opaque cells are more suited to skin infection, while white cells appear more stable in a systemic mode of infection [8]. The two types of cells also differ in their mating behavior; while opaque cells of opposite mating types readily mate, white cells do not. Indeed, white-opaque switching is itself controlled by the mating type locus- **a** and α cells can undergo switching but **a**/α cells cannot. Finally, approximately 400 genes are differentially regulated between white and opaque cells; although a few of these genes make conceptual sense (for example some mating genes are upregulated in opaque cells), most do not and, instead, point to our incomplete understanding of the fundamental differences between these two types of cells [for reviews see 24], [25].

In the present study, we examined whether white and opaque cells differ in the extent to which they are phagocytosed by cells derived from the innate immune system. We chose this property because phagocytosis of microorganisms is an especially important component of the immune response [26]. We show that two distinct cell lines derived from the innate immune system, *D. melanogaster* S2 cells and *M. musculus* RAW cells, phagocytose white *C. albicans* cells much more efficiently than they do opaque cells. This difference is seen in both the percentage of S2 or RAW cells that have phagocytosed at least one *C. albicans* and by the average number of *C. albicans* phagocytosed per S2 or RAW cell. These results are statistically highly significant and repeated in multiple assays in multiple strain backgrounds carried out over a period of six months. Experiments carried out with mixed white and opaque *C. albicans* populations produced similar results: from this mixture, S2 and RAW cells both selectively phagocytosed white cells. Taken together, these results suggest that the difference in phagocytosis is due to an intrinsic difference between white and opaque cells. For example, the mixed population experiments show that white cells do not secrete a signal that stimulates phagocytosis of opaque cells. This result is consistent with the results seen by Behnsen *et al.* 2007 where the efficient phagocytosis of *Aspergillus fumigatus* did not result in an increase of *C. albicans* phagocytosis by polymorphonuclear neutrophils in conditions unfavorable to *C. albicans* phagocytosis [27]. It is worth pointing out that while opsonization of *C. albicans* cells prior to exposure to macrophages would be expected to change the overall rate of phagocytosis, it is not clear whether the distinction between white and opaque cells would be increased or decreased.

Although this is the first study to examine phagocytosis of white and opaque cells, it is among several to indicate a differential response of the innate immune system to white and opaque cells. As described in the introduction, opaque cells are more sensitive than whites to oxidative stress and white cells, but not opaque cells, secrete a chemoattractant for leukocytes. Although it may be years before we understand the molecular basis of any of the differences in the way the host deals with white versus opaque cells, it is tempting to imagine that white-opaque switching in *C. albicans* plays a role conceptually similar to the many switching systems in bacteria, one of whose roles is to present the immune system with multiple “identities.” Although many bacterial pathogens typically use programmed DNA rearrangements to generate these differences, *C. albicans*- a eukaryote- has evolved an epigenetic mechanism that may serve the same basic function.

## Materials and Methods

### Strains

The *C. albicans* strains used for these experiments were all derivatives of CAF2-1 (URA3/ura3::λimm434). The white **a**/α strain was CAF2-1. Construction of the sorbose selected CAF2-1 **a**/**a** white (RZY12) and opaque (RZY491) strains is described in Zordan *et al*., 2006 [22]. Equivalent α/α strains were sorbose selected and derived from the same parent strain. All strains were plated on synthetic complete medium complemented with 2% glucose and 100 µg/mL Uridine (SD+aa+Uri) at 25°C and grown in liquid SD+aa+Uri at room temperature prior to assays.

### Cell Lines

*D. melanogaster* S2 cells were cultured in Schneider's medium (Invitrogen, Carlsbad, California, United States) supplemented with 10% Heat Inactivated fetal bovine serum (FBS), penicillin, and streptomycin (pen/strep)(.1 mg/mL, 100 U/mL). *M. musculus* RAW 264.7 cells were cultured in RPMI 1640 Media (UCSF Cell Culture Facility, San Francisco, California, United States) supplemented with 10% Heat Inactivated/Refiltered FBS, 10 mM HEPES, .11 mg/mL Sodium Pyruvate, .002 M L-glutamine, and pen/strep (.1 mg/mL, 100 U/mL). Jeff Cox and Erik Lontok (UCSF, San Francisco, California, United States) kindly provided the RAW 264.7 cells.

### S2 Phagocytosis Assays

The phagocytosis assay was based on the method described in Stroschein-Stevenson *et al*., 2006 [15]. Briefly, *C. albicans* liquid cultures were started from single colonies and grown overnight at room temperature. Cultures were diluted back to OD 0.2–0.5 then allowed to grow back to OD 1.0 at room temperature. 8×10^4^ S2 cells per well were plated in 96 well plastic tissue culture plates. In each well, conditioned media was added to the cell containing solution to bring the volume to 75 µL, to which 75 µL fresh media was added. *C. albicans* cultures were PBS washed and 50 µL of 8×10^6^ cells/mL PBS stock was added to each S2 well for a MOI of 5. For mixed white-opaque assays, the stock had 4×10^6^ cells/mL of each type for MOIs of 2.5 each for a total MOI of 5. Incubations proceeded at 25°C for 30 min, 1 or 2.5 hours. Cells were then transferred to Concanavalin-A coated glass bottom 96 well microplates (Greiner Bio-One, Longwood, Florida, United States) and incubated for a further hour (or 30 minutes for 1 hour time points). Fixing, staining, and examination are described below. All cell concentrations were determined by hematocytometer.

### RAW Phagocytosis Assays

RAW cell phagocytois assays were based on the S2 cell procedure with the following modifications. 4×10^4^ RAW cells were directly plated onto untreated 96 well glass bottom plates and allowed to settle for 18 hours at 37°C and 5%CO_2_. Prior to the experiment, old media was aspirated off and 150 µL fresh media was added. *C. albicans* stocks were made at 4×10^6^ cells/mL for a MOI of 5, or 2×10^6^ cells/mL of both whites and opaques for mixed culture assays. Incubations were 1 hour at 37°C and were not transferred to new wells before fixing, staining, and examination as described below.

### RAW Adherence Assays

RAW cell adherence assays were based on the RAW cell phagocytosis procedure with the following modifications. 1.2×10^5^ RAW cells were plated into the 96 well plates, and allowed to settle overnight. Following addition of fresh media, the tray was incubated for 30 minutes at 4°C. *C. albicans* stocks were made at 1.2×10^7^/mL for a MOI of 5. Incubations were for 2 hours at 4°C, after which wells were washed 4 times with PBS before formaldehyde fixing cells. Cells for scoring were not further stained. Additional control wells were stained using the normal procedure described below, phagocytosis levels in these wells decreased to almost zero for both whites and opaques. Scoring was conducted using a Zeiss Axiovert 200M microscope (Carl Zeiss, Oberkochen, Germany), the total number of *C. albicans* cells in a field of vision was manually determined for at least three distinct spots in at least three distinct wells for each condition. Assays were performed on two distinct days.

### Immunofluorescence and Microscopy

After incubations, media was removed and wells allowed to air dry for 2 minutes. Wells were fixed for 5 minutes in a 1% Formaldehyde/PBS solution, washed once with PBS, and incubated for 1 hour in PBS plus 5% FBS. Unphagocytosed *C. albicans* was detected with a 4°C overnight exposure to 1∶2000 dilutions of primary rabbit anti-Candida (Biodesign, Saco, Maine, United States, Cat# B65881). Secondary incubation was with a 1∶2000 dilution of Cy3-labeled goat anti-rabbit antibody (Jackson ImmunoResearch, West Grove Pennsylvania, United States) for two hours at room temperature. Two PBS washes were performed after each incubation. As phagocytic cell walls had not been permeabilized at this point, the antibodies could only bind unphagocytosed *C. albicans*. A 15 minute incubation with DAPI in PBS/0.1% Triton X-100 solution allowed for DNA visualization to localize S2 or RAW cells. Wells were placed in Fluoromount-G (Southern Biotech, Birmingham, Alabama, United States) for storage pending examination. Wells were scored using a Zeiss Axiovert 200M microscope (Carl Zeiss, Oberkochen, Germany). *C. albicans* cells that did not light up with Cy3 were considered to have been phagocytosed. Filaments were not scored for either assay. Batches of 100 S2 or RAW cells were scored for phagocytosis of one or more *C. albicans* cells and for the total number of *C. albicans* cells phagocytosed. At least 2 sets of 100 cells were scored for each of at least two wells from each of at least two experiments conducted on different days for each condition. Each experiment was thereby repeated at least 4 times. The number of *C. albicans* cells phagocytosed per S2 or RAW cell scored, referred to as the phagocytic index in the figures, was calculated by dividing the total number of *C. albicans* cells phagocytosed by the number of S2 or RAW cells scored, 100. Charts in this paper represent the average of 4 or 6 data sets from the multiple wells for each condition on a single day; error bars represent the standard deviation. Statistical significance of the difference between white and opaque **a**/**a** strains was determined using the t-test assuming unequal variations data analysis function in Microsoft Excel (Microsoft Corporation, Redmond, Washington, United States), setting a p-value of less than .001 for the one-tailed distribution as the significance threshold. Charts were prepared in Microsoft Excel and edited in Adobe Illustrator. Images for this paper were processed automatically using AxioVision software (Carl Zeiss, Oberkochen, Germany). The black and white images for the different color channels were automatically converted to grey (DIC), Orange (Cy3) and Blue (DAPI). Figures were assembled in Adobe Photoshop (Adobe Systems, San Jose, California, United States).
